# 1443. Nontuberculous Mycobacterial Cervicofacial Lymphadenitis Therapy: Experience from a Tertiary Care Pediatric Center

**DOI:** 10.1093/ofid/ofac492.1271

**Published:** 2022-12-15

**Authors:** Anh N Vo, Prachi Singh, Manjiree Karandikar, Kristina Rosbe

**Affiliations:** UCSF Benioff Children's Hospital Oakland, Berkeley, California; UCSF Benioff Children's Hospital Oakland, Berkeley, California; UCSF Benioff Children's Hospital San Francisco, San Francisco, California; UCSF Benioff Children's Hospital San Francisco, San Francisco, California

## Abstract

**Background:**

Nontuberculous mycobacterial (NTM) infections commonly present as a unilateral, subacute cervical lymphadenitis in immunocompetent children under the age of 5 years. The natural history of NTM lymphadenitis without intervention is slow resolution, and there is a high risk of spontaneous drainage through the skin, often result in a scar. For NTM lymphadenitis, the gold standard is complete surgical excision as it is often curative and limits scar formation. However, surgical intervention, especially complete surgical excision, carries the risk of damage to the facial nerve in the parotid and submandibular region. Antibiotic therapy is thought to interrupt the natural progression of the disease.

**Methods:**

We conducted a retrospective chart review and reviewed >700 patients, who had a diagnosis of cervical lymphadenopathy and/or atypical mycobacterial based on ICD codes. Patients received a diagnosis of NTM if they had clinical adenitis with NTM detected on culture and/or PCR, acid-fast bacilli identified, or histological findings consistent with mycobacterial infection.

**Results:**

A total of 17 cases of NTM were identified. The median age was 27 months; 52% were male and 47% were female. The median time of adenopathy prior to presentation to ENT or ID specialist was 2 months. The average size of the involved lymph node was 2.05 cm. Two patients were treated with only medical management, 6 with surgical management, 8 with both, and 1 had observation alone as patient was near resolution. Of the patients on antibiotics, a total of 4 patients had adverse effects (abdominal discomfort, diarrhea, vomiting, and/or rash). Surgical interventions include FNA, incision and drainage, curettage, and complete excision. Four patients required 2nd intervention. One patient had concerns for sinus formation but resolved in < 1 month. No patients had facial nerve palsy or hypertrophic scarring.

**Conclusion:**

Regardless of management strategy, all children in this study had resolution of NTM. Though there is a consensus that complete surgical resection is the gold standard, we demonstrate that medical management alone (anti-microbial therapy or “wait-and-see” method) can be an alternative first-line treatment. Limitations include retrospective nature of study which is limited by data available in the EMR.

**Disclosures:**

**All Authors**: No reported disclosures.

